# Croton Chloral Hydrate

**Published:** 1874-01

**Authors:** 


### MISCELLANEOUS.

### Croton-Cbloral Hydrat.

The profession and the public are chiefly indebted to Dr. Oscar Liebreich for the introduction of chloral hydrate; and this obligation is further increased by the addition of croton-chloral hydrat, which will doubtless prove an equally valuable therapeutic agent. It is of the greatest service in cases of nerve-pain. Every sufferer from neuralgia is anxious to obtain speedy relief from pain; this may be obtained by taking croton-chloral hydrat, and then the antecedent causes of the neuralgia may afterwards be enquired into and treated accordingly. The following cases are interesting, as showing the immediate relief from pain that this drug affords.

A. suffered from facial neuralgia of a most severe character; it affected her hearing and eyesight. She could not rest or take food. She took one grain of croton-chloral hydrat every hour. In three hours, she was considerably better. After taking three more doses, she was entirely free from pain.

B. suffered much from facial neuralgia dependent on decayed teeth, and had not been able to take food or sleep for three days. She was order croton-chloral hydrat in grain-doses every hour, and obtained great relief after two doses. Six doses removed the pain completely. She slept that night.

C. This patient suffered from concussion of the spine caused by a railway accident some years ago. She had every variety of treatment for the pain she suffers in the spine and the nerves proceeding therefrom. She took potassium bromide gr. 20, and croton-chloral hydrat gr. 1, three times a day, with marked relief and no bad symptoms.

E. This is a young dyspetic and neuralgic patient, and suffers greatly from dysmenorrhœa. She took two-grain doses when the paroxysms of pain came on, with marked relief.

F. has been under treatment for various neuralgiæ for some years. She has had, at one time or another, almost every external therapeutic agent in the *Pharmacopœia*—strychnia, iron, quinine, ammonium, chloride, aconite, belladonna, iodine, bromine, blisters, hypodermic injections, galvanism, together with baths and other hygienic appliances, including change of air. In this case, two grain-doses of croton-chloral hydrat, every hour afforded more speedy relief from pain than any of the above remedies. After taking eight grains, she was almost free from pain.

In thirteen patients who have taken croton-chloral hydrat, not a single bad symptom has been observed In grain-doses, it relieves pain quickly ; causes natural sleep ; no subsequent headache or furred tongue. In several cases, it acted as a gentle laxative.—Dr. Baker, in *Brit. Med. Journal.*—*Canada Lancet.*

